# Training of volunteer nurses during the Spanish Civil War (1936–1939): A historical study

**DOI:** 10.1371/journal.pone.0261787

**Published:** 2021-12-31

**Authors:** María López, Rubén Mirón-González, María-José Castro, José-María Jiménez

**Affiliations:** 1 Faculty of Nursing, University of Valladolid, Valladolid, Spain; 2 Faculty of Medicine and Health Sciences, University of Alcalá, Alcalá de Henares, Madrid, Spain; Murcia University, Spain, SPAIN

## Abstract

**Background:**

The Spanish Civil War (1936–1939) is an example of a historic event involving nurses, with the participation of professional and volunteer nurses from Spain and other countries. In this context, nurses were trained over short periods of time and recruited to work at hospitals serving the two warring camps.

**Objectives:**

To identify the characteristics of the training received by volunteer nurses on both sides in the Spanish Civil War and compare it with previous experiences in the history.

**Design:**

Historical research.

**Methods:**

Heuristic and hermeneutical analysis of nurse training manuals and news articles from 1936 to 1939. Spanish primary sources were consulted at the Red Cross Documentation Centre Archive in Madrid, the General Military Archive in Ávila, the Municipal Newspaper Archive in Madrid, and the archives of Spanish daily newspapers *ABC* and *La Vanguardia*. The following variables were analysed: duration, entry requirements, and theoretical content of the training courses. Consolidated Criteria for Reporting Qualitative Research (COREQ) has been used.

**Findings:**

Both sides in the conflict offered a varied training programme, which was supported by official institutions and private initiatives. The courses lasted between one week and two months. Entry requirements were influenced by education level, age, moral conduct, health status, and social and political background. Training content focused on the techniques needed in conflict settings and covered specific moral values.

**Conclusions:**

Despite the different social and political characteristics of the two warring factions, the variety of training programmes on offer, the entry requirements, and the theoretical content of volunteer nurse training were similar on both sides. At the end of the Spanish Civil War, volunteer nurses on the Republican side suffered reprisals or had to go into exile. We now know that some countries involved in World War II provided training courses for volunteer nurses. It would therefore be interesting to ascertain whether Spanish volunteer nurses contributed their experience to these courses.

## Introduction

Like any other social or health emergency, armed conflict has shaped the role played by nurses in healthcare delivery [1], forging a link between war and nursing. In the context of modern nursing, Florence Nightingale’s leadership in medical and healthcare provision during the Crimean War (1853–1856) is a good example of this link [2].

Throughout the 20th century, nurses participated in a number of armed conflicts around the world, tackling physical, psychological, and clinical problems. Training for civilian nurses differs in a number of ways from specific training allowing nurses to work in military hospitals, where specific preparation for ethical and care dilemmas is vital [3]. Research such as Elliot’s study of the work carried out by nurses in Iraq and Afghanistan has analysed the impact of care provided by military nurses to seriously injured soldiers in the phase following the military deployment, when psychological and social issues emerge [4].

Nurse training is crucial in preparing nurses for the challenges of working in a conflict setting, where their theoretical knowledge, ethical commitment, and social responsibility are put to the test as they provide care in hostile, unpredictable, and challenging environments [5]. Public health crises, natural disasters, and wars are unexpected events that can take place at any time. In the 21st century, the COVID-19 pandemic is one such event, involving nurses directly in an unprecedented health crisis [6]. In response to this situation, a number of countries have been obliged to recruit student nurses to work in field hospitals set up to respond to the pandemic [7, 8].

Public health emergencies have shaped the development of the nursing profession, imbuing it with the social, political, and economic values of each historical context and influencing and modifying its image over time. It is important to study these emergencies and their incorporation into training programmes in order to understand nurses’ influence and contribution to society [9].

## Background

At the beginning of the 20th century, the professional practice of caregiving was segregated by gender [10]. For the male nurse, Nightingale’s reform was a professional brake [11], while for the female nurse it was an educational and professional opportunity [12]. In the case of Spain, we find the figure of the nurse and the *practicante* (practitioner). The nurse was a professional related to the woman-caregiver-salaried duality and the practitioner with the male-healer-autonomous duality [11].

The Spanish Civil War (1936–1939) is one example of a historic event involving nurses, with the participation of professional and volunteer nurses from Spain and other countries. During the war, nurses brought their knowledge, academic training, and professional experience to bear [13] to provide the best possible healthcare on both the Republican side, the part of the country that supported the democratic government established by the Second Spanish Republic (1931–1936), and the Nationalist side, which supported the coup d’état led by General Francisco Franco-Bahamonde (1892–1975) in July 1936 [14].

The mobilisation of nurses by both camps was crucial in managing healthcare during the conflict. Military hospitals called for nurses to cover shortages as they were overwhelmed by rising numbers of sick and wounded patients [15]. One of the medical institutions that helped the Military Health Corps to provide care and address staffing shortages was the Spanish Red Cross. When the war broke out, Spain did not have enough qualified nurses to meet healthcare needs, so both sides began to use training courses to equip women with the skills needed to work as volunteers in hospitals. The majority of volunteer nurses were Spanish women from different social backgrounds who were keen to help during the conflict. Fewer women of other nationalities volunteered to work as nurses in Spain [16].

This system was used in both Republican and Nationalist areas in the Spanish Civil War. In both camps, professional and volunteer nurses had different caring responsibilities and types of work. The Republicans received support from largely professional nurses from the International Brigades and the International Red Aid, most of whom were qualified and had professional experience in their country of origin [17, 18]. When they arrived in Spain, the nurses from the Brigades worked in field hospitals with volunteers from areas near the front line and oversaw the provision of care [19]. Meanwhile, statements from foreign volunteers such as Priscilla Scott-Ellis, who volunteered to help without any nursing experience or training, have been found on the Nationalist side [20]. British Quakers also provided humanitarian aid on both sides, although they appear to have been more aligned with the Republican side [21].

In terms of historiography, we find some researches that have identified the training of volunteer nurses during World War I (1914–1918) [22], the Finnish Civil War (1918) [23] and the World War II (1939–1945) [24, 25]. However, none of them provided information of the characteristics of the training. Regarding the Spanish Civil War, we have some testimonies of women who were trained in this training courses for volunteer nurses on both sides [26, 27] and we know that manuals from professional nursing schools were used [28]. However, we know very little about the characteristics of the training courses offered by both sides and the *ad hoc* manuals. Therefore, the aim of this study is to identify the characteristics of the training provided to volunteer nurses on both sides of the conflict during the Spanish Civil War (1936–1939) and to compare it with previous experiences in the history.

## Materials and methods

The study used a heuristic, hermeneutical historical method based on document analysis of primary historical sources. The hermeneutical method is used to interpret historical texts. It is not an intuitive process, as it requires researchers to remain vigilant to developments in the phenomenon under analysis while selecting appropriate sources [29].

With regard to the selection of historical sources, it is important to note that the first works published during the Spanish Civil War and the first few decades of the Franco regime were shaped by ideology and propaganda from the opposing sides [30]. It is difficult to find sources from the Republican faction, as it was forced to destroy all its documents to avoid reprisals after losing the war. Moreover, as the war progressed, the Republicans were in constant retreat while the Nationalists became more consolidated, contributing to the loss of relevant documents. This was compounded by the Pact of Forgetting (*Pacto del Olvido*) at the beginning of the Spanish democracy (1975-) [31]. It is important that more in-depth analysis of the healthcare situation in the Republican zone is carried out to allow a more precise understanding of the training received by nurses working on this side of the conflict. Limited access to Republican sources is the main limitation of this study. Taking these archival constraints into consideration, nurse training manuals and historic news articles on training courses for war nurses from the Republican and Nationalist zones dating from 1936 to 1939 were included in the study.

The training manuals analysed, used in nursing schools and training courses, were generally written by doctors, one of whom was Manuel Usandizaga-Soraluce (1898–1982) [32], Professor of Obstetrics and Gynaecology. Doctor Usandizaga-Soraluce managed the Casa de Salud Valdecilla in Santander, Cantabria, Spain, one of the most important nursing schools in the early professionalization of nursing in Spain [33]. Only one manual written under supervision from the Red Cross Medical Teaching Commission [*Comisión Médica de Enseñanza de Cruz Roja*] and translated from the French by María de Corral, a nurse with the Spanish Red Cross, was available [34]. These manuals are available in the Red Cross Documentation Center Archive and in the National Library of Spain, both located in Madrid.

In terms of documentary sources the researchers consulted the personal correspondence of Mercedes Milá y Nolla, Inspector of the Female Military Health Corps and the ultimate head of the training of volunteer nurses on the Nationalist side, to the different nursing supervisors of regional military hospitals. This correspondence is available in the General Military Archive in Ávila and cited at the end of the manuscript. No archive documents of this kind were found on the Republican side. To safeguard against this possible bias, historical press and legislative documentation from both warring geographical areas have been consulted. This is the case of the newspaper *ABC* published in the city of Seville, National side, and *La Vanguardia* published in the city of Barcelona, Republican side. The historical press was consulted in the Municipal Newspaper Archive in Madrid, and the legislative documentation in the database Gazeta [35]. Texts that referred solely to official, formal training delivered at nursing schools and did not focus on courses for volunteer nurses were excluded from the study.

The following variables were analysed: types of training courses, duration, entry requirements, and theoretical content of the courses. The variables were determined inductively, i.e. first the sources were found and then it was decided to group the results according to the area of interest [36]. If we want to apply rigour in historical research, we have to keep an open mind and let the historical evidence speak [37].

The researcher ML collected the archival sources from Madrid and Ávila. The variables were analyzed by the four authors, all of them are PhD. ML and RMG are registered nurses and have completed historiographical doctoral theses. RMG has participated in various historiographical national research projects. Half of the researchers are women. In the moment of the study, all four researchers are lecturers in faculties of Nursing.

Considering that historical research is a qualitative methodology [37], the Consolidated Criteria for Reporting qualitative Studies (COREQ) checklist has been applied [38]. However, some elements of COREQ checklist are not applicable in historiographical studies.

## Findings

### Types of training courses

Training courses for volunteer nurses were provided by both sides throughout the conflict. Some of these courses were private initiatives, while others received support from official institutions, giving rise to a wide range of training opportunities with the same purpose: to equip auxiliary personnel with the theoretical and practical knowledge required for them to provide adequate care to wounded patients.

In the Republican zone, the Red Cross organised courses to train war nurses in the cities of Barcelona, Madrid, and Murcia [39]. The Directorate of Health also organised nurse training courses to meet the need for this type of personnel at military hospitals [40].

In the Nationalist zone, the professional landscape was highly varied due to the range of different training courses offered by the organisations that were responsible for managing the mobilisation of women during the war. Besides the courses delivered by the Red Cross, the training provided by military hospitals allowing women to obtain the Military Health Auxiliary Nurse certificate, which was required to work on the front lines and in military medical centres, had the greatest uptake [41]. For the most part, these courses were taught by doctors, with support from professional nurses and the Daughters of Charity in practical lessons [42].

### Duration of training courses

The duration of the training courses depended on the type of course and the institution responsible for delivering it. In the Republican zone, the Ministry of Health organised training courses at the Nursing School at the Faculty of Medicine in Valencia, which were initially one month long but were extended to three months. Practical classes were held from 09:00 to 13:00 and theory classes from 15:00 to 18:00 [43]. During the first few months of the war, the demand for nurses prompted the organisation of several week-long courses to provide “the most basic knowledge involved in caring for the wounded, as well as the simplest clinical and laboratory practices” (All quotes have been translated by the authors) [44].

In the Nationalist zone, the duration of the training on offer also varied from course to course. The courses provided by the Military Health Corps lasted two months and participants were required to pass an exam in order to obtain the certificate and licence accrediting their training and allowing them to start work [45, 46]. Theory classes were usually scheduled in the evenings from 18:00 to 20:00, from 18:00 to 19:00, or from 19:00 to 20:00, depending on the hospital. Practical classes were held in the mornings from 10:00 to 13:00, with trainees helping at the hospital as they learned [47–49].

In the Nationalist zone, some courses were limited to 30 working days to avoid “tiring and discouraging” volunteers, and each lesson lasted 45 minutes [50]. Other courses, where discipline and responsibility were key, had a minimum duration of four months [51]. Universities also joined the initiative, offering nursing courses with an approximate duration of 30 days, after which trainees had to pass an aptitude test in order to obtain an accreditation certificate [52–54].

### Entry requirements

In the Republican zone, the courses held by the Ministry of Health at the Faculty of Medicine in Valencia were open to 40 women at a time, who were selected by the healthcare unions. The best applicants were selected on the basis of their level of general knowledge, although they had to be aged between 18 and 35. The Nursing School was responsible for selecting the final participants using an aptitude test [55].

In the Nationalist zone, the Military Health Corps required applicants to accredit their good moral and social conduct. They and their families were also expected to have the right political background [41]. In the case of the courses offered by political organisations, participants were required to be activists from the organisation [56]. The maximum number of trainees was 40, while the minimum age for admission was 20 and the maximum age was 40. Despite the rules, the reality is that they received younger students, as one of Milá’s letters indicates:

“You are sending me a little girl that you have allowed to sit for the Auxiliary exams [volunteer nurses] despite the fact that the rules state that the minimum age is 20 years old and she is 16. It’s nonsense that these girls hang around in hospitals instead of school, so they can’t be given the [training] card! Not being mobilised or on night shift, we cannot be indulgent until 18, but not less in any way. I don’t know what these parents are thinking about!” [57].

Continuing with the Nationalist zone, applicants were asked to submit a certificate of good conduct, their birth certificate, and their academic qualifications with their application [58]. In other courses, trainees were told that discipline and responsibility were essential so they must attend all classes and make sure to arrive on time. If they failed to do so, they would not be allowed to take the final exam before the board of examiners [51].

### Theoretical content of the courses

The conflict situation led to a change in training content (see Table 1), which focused largely on caring for the war-wounded and on rapid acquisition of the basic knowledge needed to help them. Knowledge of different types of gas and treatments for gas poisoning [59], as well as emergency care and first aid, became essential components of nurse training manuals.

**Table 1:** Content of nurse training manuals [32].

NEW TRAINING CONTENT				
Bruises and wounds	Wound infections	Sterilisation	Surgical wound care	Dressings
Bleeding	Fractures, sprains, and dislocations	Injuries caused by heat or cold	Caring for the wounded in hospital	Wound and surgical complications

As well as new training manuals focusing on knowledge relating to the conflict situation, some existing manuals also added sections for this purpose, including nursing, chemical warfare, and military administration [34].

Theory lessons covered the spirit of sacrifice, sense of duty, caution, patience, and charity [60]. Volunteers were required to be in an excellent state of health and lead a “hygienic life, with a good diet, physical exercise, and regular sleeping times” [61] to ensure that they were able to cope with the work and deliver healthcare to patients [62]. In the manuals, trainees were urged to pay careful attention to their personal hygiene to avoid spreading disease, among other reasons. The use of uniforms, the careful cleaning of these uniforms, and washing of parts of the body that were not covered by the uniform but came into contact with the patient were particularly important [61].

Training manuals also addressed matters relating to public perceptions of nurses, encouraging specific moral qualities. Their humanitarian work was expected to be characterised by kindness, gentleness, and altruism, as well as warmth, which nurses were required to display to all patients while remaining discreet and respecting patient confidentiality [61].

In the training provided for nurses on the Nationalist side of the conflict, a sense of vocation and Christian charity (values prized by the Franco dictatorship) were presented as the model of perfection that nurses should strive to attain, with no expectation of professional recognition beyond that of God. Religious indoctrination became more entrenched as the war went on and nurses were asked to profess their love of God “by practising a Christian life of meditation and piety” that would allow them to care for the sick [63].

## Discussion

The contribution of volunteer nurses during World War I is widely recognised [64], raising the visibility of women’s work in healthcare settings [65] and highlighting the importance of professional training [66]. In the case of the Spanish Civil War this recognition was given by the republican government through its different organizations, political parties and labour unions. This reality has been related by some survivors’ testimonies on both sides [26, 27]. Despite ideological differences on both sides, there was a mutual interest in women’s free labour.

Although dependence on the medical profession waned during World War I [67], doctors continued to write training manuals for volunteer nurses during the Spanish Civil War. This is the case of Dr. Usandizaga, who published two works during the Spanish Civil War for the training of volunteer nurses, but only 13% of the content was unpublished with respect to his previous editions [28].

In World War II, the countries involved were already equipped with professional nurses, albeit in insufficient numbers. Nurses had to be mobilised from different countries [68, 69], students without clinical experience were recruited [70], and volunteer nurses were trained [24, 25]. The practice of volunteer nurse training was already in place before World War II; however, it was used during the Finnish Civil War (1918) [23] and in the Spanish Civil War.

During the Spanish Civil War, training courses lasted for a short period of time as their purpose was to provide intensive training for volunteers, including World War II volunteer nurses from Brazil [25] and Finland [24]. This training was required to allow the volunteers to work as nurses in military hospitals, first aid stations, medical trains, pharmacies, and prisoner-of-war hospitals [24].

We have identified that although the courses provided by both sides in the Spanish Civil War varied in terms of the training provided, their duration, and their entry requirements, the theoretical content was similar in all of them. The aim was to prepare volunteer nurses to provide healthcare in conflict settings. In such settings, knowledge and professional skills are extremely important. Studies such as Firouzkouhi et al. provide evidence of this, focusing on the need to include specific training in triage to ensure adequate healthcare [71].

As we have seen, comprehensive, targeted training was needed during the Spanish Civil War to meet healthcare needs. The volunteers’ lack of experience and training were compensated by their eagerness to help and their good intentions, but these were not always sufficient to enable them to cope with the emotional distress caused by working in a war zone. None of the lessons in the manuals referred to the pain and suffering that the nurses would witness. They were asked to be kind, pious, and caring, but they were not prepared for the mental fatigue that they would face when providing compassionate care [72]. These patriarchal values were also present in the training of World War II volunteer nurses from Brazil, who were required to be feminine, caring, selfless, innocent, meek, and always available [25].

The training provided by the Spanish Civil War courses contributed to the public image of what a nurse should be, emphasising the moral qualities that nurses were expected to possess in particular. As well shaping their image, training courses also helped to foment a sense of belonging to a group and served as a way of homogenising attitudes and practices, as well as highlighting gender issues in this context [73]. In this respect, the press played a major role in recruiting female volunteers, including Brazilian volunteer nurses [25].

We have not found any cases of training courses for male nurses (practitioners) during the Spanish Civil War. This is related to the sexualisation of care that was present in the early 20th century [10]. It would be interesting to analyze whether there were any training courses for male volunteer nurses in war contexts and the possible differentiation or similitudes with the female training courses in the future.

As well as theory lessons on providing healthcare and assistance to the wounded, the training manuals also contained recommendations on rest, diet, and exercise for nurses to ensure that they remained healthy and able to carry out their work to the best of their abilities. Recent studies have demonstrated the importance of self-care among nurses. Clinical training, rest, recuperation, exercise, and diet are all important factors in reducing psychological stress in war zones [74].

During the Spanish Civil War, volunteers nurses were trained in the ethics of care, twinned with vocation and humanitarianism, which are essential in any conflict setting [75]. Both sides of the conflict used the idea of vocation to mobilise nurses [76], with the Nationalists justifying it on the basis of Christian values and the Republicans on the basis of political and democratic values. The idea of vocation is influenced by the politicisation of training and a similar phenomenon can be observed among medical volunteers in the Finnish Civil War (1918) [23].

The outcome of the Spanish Civil War and the first few decades of the Franco dictatorship (1939–1975) shaped the development of the nursing profession in Spain. The dictatorship revoked nursing diplomas issued by the Republican side [15], retaliated against Republican nurses [77], and supported an education system based on the values of the regime, including discipline and the spirit of sacrifice [78]. Professional and volunteer nurses who opted not to live in fear and to continue to fight for democratic values were forced into exile [79].

Under the 1944 Spanish Health Law (*Ley de Sanidad*), nursing, midwifery, and nursing assistant studies were unified in Spain in 1953 under a new qualification named *ayudante técnico sanitario* (technical health assistant) [80]. For decades, the training of technical health assistants in Spain fell far short of the recommendations of the World Health Organisation [81] and focused on providing doctors with assistants as part of a technical approach to healthcare provision. Nursing studies were incorporated into Spanish universities in 1977 following the advent of democracy (1975-), bringing about an important change in nursing curricula with the inclusion of new modules such as nursing fundamentals [82].

## Conclusion

The health emergency caused by the Spanish Civil War gave rise to a need for greater numbers of nurses, leading to the emergence of courses aiming to train volunteers on both sides in a pattern that would be repeated in World War II years later. The variety of training programmes, entry requirements, and theoretical content were similar in both factions, although they were shaped by different sociopolitical contexts. The outcome of the war was pivotal in the lives of the volunteers. Volunteer nurses on the Republican side went into exile, so it is difficult to know how their experiences in the Spanish Civil War may have influenced volunteer nurse training systems in World War II or their integration into the workforce in their destination countries. The volunteer nurses on the winning Nationalist side helped to consolidate the nursing profession and nurses’ professional identity, which were shaped by the values of the Franco dictatorship.
